# Heme oxygenase-1 in protozoan infections: A tale of resistance and disease tolerance

**DOI:** 10.1371/journal.ppat.1008599

**Published:** 2020-07-21

**Authors:** Rafael C. M. C. Silva, Leonardo H. Travassos, Claudia N. Paiva, Marcelo T. Bozza

**Affiliations:** 1 Laboratório de Inflamação e Imunidade, Departamento de Imunologia, Instituto de Microbiologia, Universidade Federal do Rio de Janeiro, Rio de Janeiro, Brasil; 2 Laboratório de Imunoreceptores e Sinalização, Instituto de Biofísica Carlos Chagas Filho, Universidade Federal do Rio de Janeiro, Rio de Janeiro, Brazil; Boston College, UNITED STATES

## Abstract

Heme oxygenase (HO-1) mediates the enzymatic cleavage of heme, a molecule with proinflammatory and prooxidant properties. HO-1 activity deeply impacts host capacity to tolerate infection through reduction of tissue damage or affecting resistance, the ability of the host to control pathogen loads. In this Review, we will discuss the contribution of HO-1 in different and complex protozoan infections, such as malaria, leishmaniasis, Chagas disease, and toxoplasmosis. The complexity of these infections and the pleiotropic effects of HO-1 constitute an interesting area of study and an opportunity for drug development.

## Introduction

The control of labile heme amounts is a main asset in the daily fight for survival, especially relevant in conditions of hemolysis, extensive tissue damage, and infection. Throughout evolution, several mechanisms were selected that counteract the deleterious effects of labile heme [1, 2]. The scavenging proteins haptoglobin and hemopexin contribute to the clearance of hemoglobin and heme from the bloodstream [3]. In the intracellular environment, heme is degraded by heme oxygenases (HOs), enzymes expressed by most living organisms and coded by the highly conserved *HMOX* genes [4]. HO cleaves heme, generating equimolar amounts of biliverdin (which can be further reduced to bilirubin by biliverdin reductase), carbon monoxide (CO), and ferrous iron [5]. HO-1 is inducible, and its expression is triggered by a number of stress-inducing stimuli, while HO-2 is constitutively expressed in the kidneys, liver, testes, and central nervous system [4, 6].

Given its critical role in homeostasis [7–10], it is not surprising that HO-1 profoundly affects the host response to infection in multiple ways [4, 11–13]. There are 2 main ways that a host deals with infection; one is through resistance, the ability to restrict or eliminate the infectious agent, a function primarily performed by the immune system. The second, disease tolerance, is the capacity of the host to mitigate or avoid the pathological consequences of an infection [14, 15]. Several pieces of evidence indicate that HO-1, by reducing labile heme amounts or dependently on the products of heme catabolism, can interfere with resistance and disease tolerance. In fact, the induction of HO-1 by inflammatory stimuli, including heme, proinflammatory cytokines, and pathogen-associated molecular patterns (PAMPs), indicates that HO-1 is part of a negative feedback loop controlling inflammation and tissue damage during infection [4, 16–18].

Heme is a potent inducer of oxidative stress and inflammation, thus upon infection, it can contribute to cell death and tissue damage [19–25]. Besides influencing tissue tolerance, heme could also impair the resistance to infection through the inhibition of phagocytosis and as a nutrient that facilitates pathogen growth [26, 27]. A recent study, however, indicates that heme can promote innate immune memory, increasing resistance to bacterial infection [28].

By strongly inducing HO-1, heme triggers its own catabolism as a feedback control system. HO-1 reduces the amounts of free heme and generates the antioxidant products biliverdin-bilirubin and CO, thus changing the redox balance that influences disease tolerance and resistance [29–31]. Several studies support that biliverdin and bilirubin generation is an important part of the tissue protective antioxidant role associated with HO-1 [32–37]. CO is also an antioxidant with cytoprotective effects that induces the anti-inflammatory cytokine interleukin 10 (IL-10), inhibits the production of inflammatory mediators, and binds to heme on the hemoglobin avoiding heme release [38–41]. Biliverdin, bilirubin, and CO also have microbicidal activities, contributing to resistance against infection [42–45]. Interestingly, CO triggers ATP release by bacteria, which in turn induces IL-1β production dependently on NLRP3, macrophage activation, and bacterial killing [46].

HO-1 not only contributes to a differential susceptibility of the cells to stress but also alters the iron pool availability to pathogens through the release of iron from heme and by inducing the expression of iron scavengers and transporters [47–48]. In fact, many of the protective effects mediated by HO-1 are linked to ferritin H chain (FtH), which forms a protein complex with crucial cytoprotective and antioxidant functions. FtH is induced by free iron and scavenges ferrous iron, preventing redox deleterious reactions and the availability of iron to pathogens [49, 50]. In some circumstances, exacerbated expression of HO-1 is associated with cytotoxic effects mediated by uncontrolled iron amounts [51].

Protozoan infections are an important cause of morbidity and are among the major causes of death worldwide [52]. In what follows, we discuss the current knowledge of the mechanisms and roles of HO-1 during infections caused by the major protozoan pathogens, using as a framework the paradigm of resistance and disease tolerance.

## HO-1 in malaria

With an estimated 228 million cases worldwide in 2018, malaria presents one of the highest morbidities and mortality rates among parasitic diseases (estimated number of deaths at 405,000) [53]. The human disease is caused by different *Plasmodium* species, including *P*. *falciparum*, *P*. *vivax*, *P*. *malariae*, *P*. *ovale*, and *P*. *knowlesi*, and is characterized by 2 distinct stages: the hepatic (or pre-erythrocytic) and the erythrocytic. In the hepatic stage, after invasion of hepatocytes and differentiation into merozoites, parasites are released into the bloodstream. Then, merozoites invade and replicate within red blood cells, leading to their lysis to initiate the erythrocytic phase, a stage associated with the release of heme into the circulation and high fever [54–56]. The severe forms of malaria include cerebral malaria (CM), severe malaria anemia (SMA), and acute respiratory syndrome (ARDS). Despite important differences regarding the human disease, mouse models constitute important tools to characterize the molecular mechanisms of malarial pathogenesis.

In 2007, Pamplona and colleagues established a clear connection between HO-1 expression and experimental cerebral malaria (ECM) in mice. Infection with *P*. *berghei* ANKA causes high rates of ECM and low expression of HO-1 in C57BL/6 mice, in contrast to the paucity of disease signs and high expression of HO-1 observed in the BALB/C strain [57]. Indeed, genetic disruption of *Hmox1* (the gene that encodes HO-1) in BALB/C mice led to an increase in the development of ECM, and conversely, the induction of HO-1 expression in C57BL/6 mice prevents pathology and lethality. Importantly, the reduction of ECM and lethality conferred by HO-1 occurs independently of changes on parasitemia, indicating that HO-1 triggers disease tolerance without affecting the resistance. The protective effect of increased HO-1 expression is mimicked by the administration of CO [57]. CO has several effects that converge to a beneficial disease outcome—including its ability to bind heme, thus reducing its release from hemoglobin, the inhibition of proinflammatory cytokines, the recruitment of leukocytes, and the tissue damage—without interfering with the parasite load. These fundamental findings were confirmed with a CO-releasing molecule (CO-RM), capable of protecting against ECM through the induction of HO-1 and without affecting oxygen transport by hemoglobin [58].

Individuals with sickle-cell trait, heterozygous with a point mutation in the β chain of hemoglobin, have hemolytic episodes and reduced incidence of the severe forms of malaria, including the cerebral manifestations and anemia [59–60]. Importantly, transgenic sickle-cell disease hemizygous mice also have episodes of hemolysis and are protected against ECM, with similar parasitemia as the controls, in a mechanism associated with disease tolerance mediated by the activity of HO-1 [61]. This notion is in sharp contrast to the accepted paradigm that attributes the protection to an increased resistance of sickle cell disease (SCD) erythrocytes to *P*. *falciparum* infection. The control of heme concentrations during acute malaria infection was associated with a milder proinflammatory response in children with the HbAS genotype compared to the HbAA genotype [62]. Surprisingly, in this study the expression of HO-1 was not associated with the reduction of heme.

HO-1 inhibits the expression of CXCL10, and conversely, upon *P*. *berghei* ANKA infection, *Cxcl10−/−* mice have increased survival despite lower levels of HO-1 protein expression, indicating a central role of the CXCL10-CXCR3 axis on HO-1 expression and ECM [63, 64]. Importantly, heme induces CXCL10 expression, contributing to CD8+ T-cell recruitment and activation in the brain microvasculature [65]. These results reveal an intricate regulatory loop involving heme, HO-1, and CXCL10 during malaria.

The protective effects of increased HO-1 expression and CO treatment have also been demonstrated in malaria-associated acute lung injury in mice infected with *P*. *berghei* ANKA [58, 66], thus suggesting that these treatments, by reducing the oxidative stress and the inflammatory response, constitute promising strategies against this important cause of death in adults with malaria [67]. HO-1 also protects mice in other experimental models of malaria, as demonstrated by the high rates of hepatic failure and death of *Hmox1*-deficient mice infected with *P*. *chabaudi chabaudi* compared to the benign outcome of BALB/C controls [68]. In line with the idea that HO-1 promotes disease tolerance in malaria, mice transduced with a recombinant adenovirus expressing HO-1 in the liver and infected with *P*. *chabaudi* have reduced inflammation and tissue damage, through an antioxidant effect that prevents hepatocyte apoptosis without affecting pathogen load. Treatment with the antioxidant N-acetylcysteine (NAC) reduces inflammation and recapitulates the protective effect of HO-1 on liver injury caused by *Plasmodium* infection [68][69]. Importantly, heme released from parasitized red blood cells induces formation of intravascular neutrophil extracellular traps (NETs) dependent on reactive oxygen species (ROS) and contributes to increased neutrophil inflammation and liver damage in an experimental model of malaria [70].

**Fig 1:**
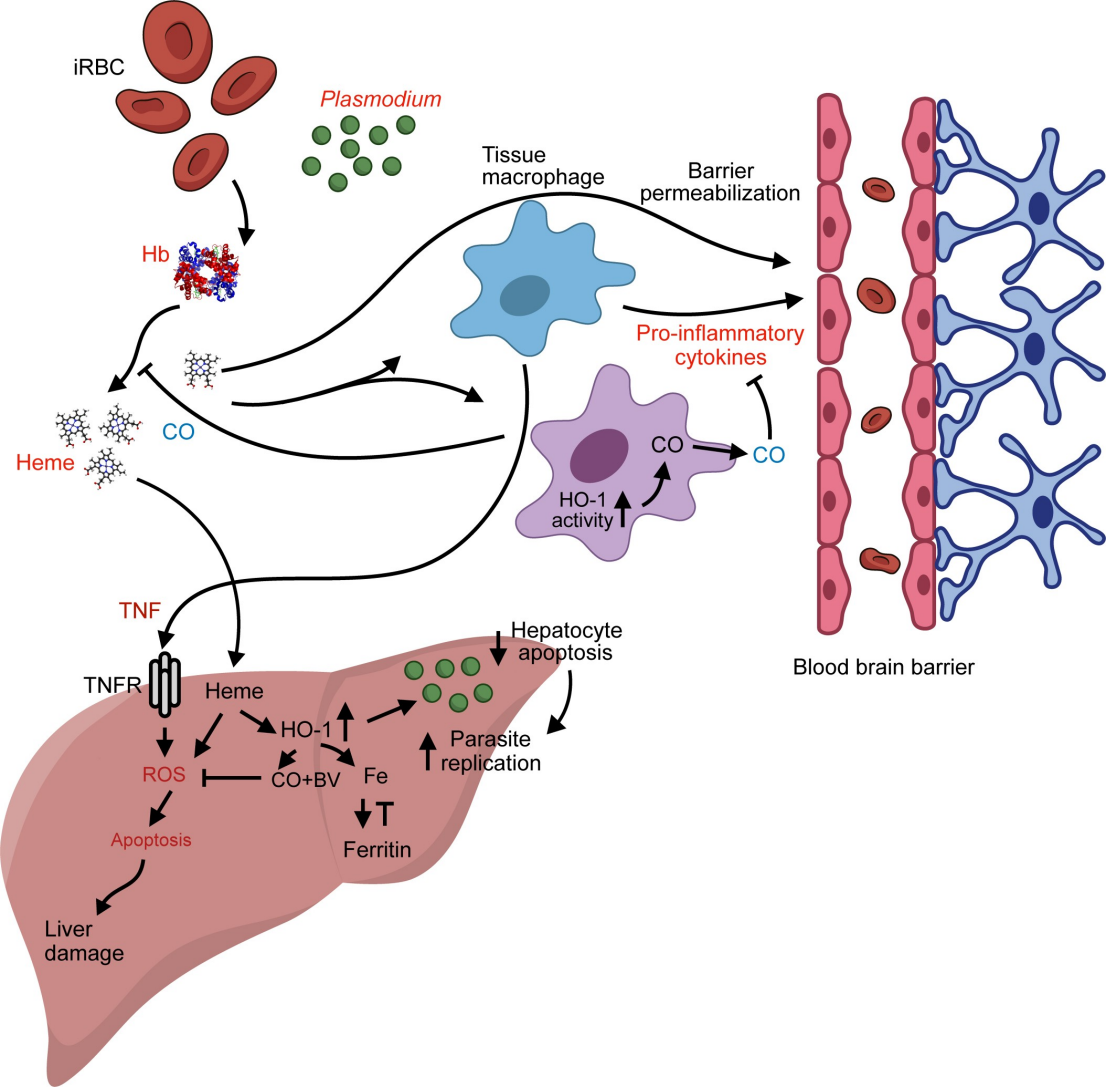
Role of HO-1 in malaria. The outcome of HO-1 expression depends on the stage of the disease. In the liver stage, HO-1 protects infected hepatocytes from dying, allowing a greater parasitism, thus reducing disease resistance to infection. During erythrocytic phase, HO-1 is crucial to prevent heme-induced inflammation through its catabolic activity. Its byproduct CO reduces heme levels by complexing with Hb and avoiding heme release. These effects combine with its immunoregulatory and cytoprotective capacity to drive the protective effects in this phase of infection, increasing the disease tolerance. BV, biliverdin; Hb, hemoglobin; HO-1, heme oxygenase 1; iRBC, infected red blood cells; ROS, reactive oxygen species; TNF, tumor necrosis factor; TNFR, tumor necrosis factor receptor.

Recent evidence supports the notion that HO-1-dependent FtH expression is required for cytoprotection against *P*. *chabaudi* infection through the inhibition of c-Jun N-terminal kinase (JNK) activation [71]. Even more striking is the observation that mice lacking the expression of HO-1 or FtH exclusively on proximal tubular epithelial cells have increased acute kidney injury and lethality in a model of malaria caused by *P*. *chabaudi*, despite similar parasitemia, compared to the infected controls [72]. These results favor the idea that HO-1 and FtH provide protection in malaria mouse models through tissue-specific disease tolerance. Conversely, increased HO-1 expression in the liver stage during the infection with *P*. *berghei* ANKA and *P*. *yoelli* in BALB/C mice has been associated with disease progression [73]. In this study, inoculated sporozoites invade, proliferate, and differentiate to merozoites inside hepatocytes. HO-1 and/or its byproducts CO and biliverdin were described to protect infected hepatocytes from cell death, which significantly increased the number of parasites that reach the erythrocytic stage of the parasite life cycle. Thus, HO-1 cytoprotective effects are associated with lower disease resistance and increased parasite load during the liver stage, exposing a putative mechanism of parasite evasion associated with induction of HO-1 expression. Thus, HO-1 can affect both resistance and disease tolerance during malaria infection (Fig 1).

Malaria is also associated with an increased mortality after secondary bacterial infections [74]. Interestingly, inhibition of HO-1 reverts the susceptibility to *Salmonella* during *P*. *yoelii* infection in mice [75]. These results suggest that the increased susceptibility is governed by HO-1 induction through mobilization of immature progenitor cells from the bone marrow. These immature cells possess defects on oxidative burst and are not able to restrict intracellular bacteria proliferation, which correlates with increased bacterial burden. It is important to mention that higher susceptibility to bacterial infections have also been described in patients with SCD, in which increased free heme circulation and HO-1 expression is expected [76]. A recent study indicates that heme can also impair bacterial clearance independently of HO-1, through dysregulated actin polymerization and inhibition of phagocytosis via Dedicator of Cytokinesis 8 (DOCK8) and Cell division control protein 42 homolog (Cdc42) activation [26]. Thus, HO-1 decreasing host capacity to deal with infections and heme through inhibition of phagocyte activity can contribute to higher susceptibility to secondary infections after hemolytic events.

The involvement of HO-1 in human malaria is a matter of intense debate. Schluesener and colleagues (2001) demonstrated the up-regulation of HO-1 in lesions of individuals with CM, although its role in protection or disease progression was not addressed [77]. In addition to the complex regulation of HO-1, in humans a polymorphism in the *HMOX1* gene affects the level of HO-1 expression upon different stimuli. This polymorphism is observed in the number of GT repeats in the promoter region of the gene. Low frequency of GT repeats is associated with a higher expression of HO-1. Takeda and colleagues explored the correlation between (GT)n polymorphism and susceptibility to CM in Myanmarese patients with uncomplicated malaria and CM and found that the frequency of homozygous for the shorter (GT)n alleles was significantly higher in CM patients than those with mild disease, thus representing a genetic risk factor for CM [78]. Similarly, *HMOX1* gene promoter alleles and SNPs associated to higher HO-1 expression correlate with severe malaria in children [79]. On the other hand, Kuesap and colleagues did not find an association between HO-1 polymorphism and disease severity in 329 cases of non-severe malaria (with acute uncomplicated *P*. *falciparum* malaria) and 80 cases with *P*. *vivax* malaria, and 77 cases with severe or CM for analysis of genetic polymorphisms of HO-1 [80]. Mendonça and colleagues (2012) described that long (GT)n repeats were associated with symptomatic malaria in a study including 264 patients with symptomatic malaria, asymptomatic malaria, and uninfected individuals [81]. Although these contrasting results could be, at least in part, explained by different etiologic agents, further studies are required in order to dissect the impact of polymorphisms in *HMOX-1* in malaria progression and severity.

## HO-1 in Chagas disease

Chagas disease is the result of the infection by the protozoan parasite *Trypanosoma cruzi*. Several strains with high variability have been associated with the characteristic morbidity of the disease, and although described as a single species, *T*. *cruzi* strains show major differences [82]. Chagas disease affects 11 million people in endemic areas of Central and South America, but some cases have been reported in Europe and North America, mainly in immigrants from these endemic areas [83]. Between 30% and 40% of infected individuals develop the chronic and debilitating forms of the disease. Two-thirds of these patients develop cardiomyopathy, and a third will develop the gastrointestinal form [84]. As for malaria, little is known about what drives the susceptibility of certain individuals to chronic phase pathology or even to a fatal progression (50% of the individuals who develop the cardiac forms die from sudden death and 37% from heart failure).

Recent studies were performed to understand the role of HO-1 in mouse models of Chagas disease. The induction of HO-1 by Cobalt protoporphyrin IX (CoPPIX) reduces parasite burden in vivo and also on macrophages in vitro [85]. These effects could be mimicked by HO-1 gene overexpression in macrophages, providing pharmacological and genetic evidence of the importance of HO-1 in this setting. However, HO-1 is clearly not the sole effector mechanism promoted by CoPPIX, since treatment of *Hmox1*^−/−^ bone marrow derived macrophages (BMDMs) with CoPPIX also reduces parasite burden. Overexpression of nuclear factor erythroid 2-related factor 2 (NRF2), a transcription factor known to control antioxidant responses (including HO-1 expression) also reduces parasite burden, while CoPPIX fails to reduce the parasite burden of infected *Nrf2*^*−/−*^ BMDMs [85]. These results indicate that NRF2 activates redundant pathways that lead to parasite control. HO-1 exerts effects on *T*. *cruzi* by different mechanisms. Since heme is an indispensable molecule for *T*. *cruzi* biology [86, 87], the biosynthetic pathway is absent in this parasite [88], and it is possible that increased HO-1 expression and consequent reduction of the intracellular heme pool is detrimental to the parasite. Reductions in the pool of intracellular labile iron are also detrimental to the parasite. It has been shown that CoPPIX along with other antioxidants, such as NAC and apocynin, increase the expression of FtH and ferroportin to affect the availability of labile iron [85]. These results suggest that ROS likely acts mobilizing iron to increase parasite growth. In fact, manipulations of the iron pool were reported to interfere with parasite proliferation, indicating that the parasite also feeds on labile iron.

Gutierrez and colleagues showed that the administration of Zinc protoporphyrin IX (ZnPPIX), a chemical inhibitor of HO-1, reduces the survival and increases the production of proinflammatory cytokines and the cardiac inflammatory infiltration in *T*. *cruzi*–infected BALB/c mice [89]. Surprisingly, although in this model the HO-1 inducer heme reduced cardiac inflammatory infiltrate and prolonged survival, both ZnPPIX and heme decreased acute phase parasitemia. These results differ from those from Paiva and colleagues, in which infected C57BL/6 mice treated with the inhibitor of HO-1 activity Tin protoporphyrin IX (SnPPIX) had increased parasite burden and inflammation [39]. Both groups worked with *T*. *cruzi* Y-strain, using different mouse lineages. The reasons for the conflicting effects between metalloporphyrins ZnPPIX and SnPPIX remain unclear. It must be acknowledged, however, that this latter work used the HO-1 inducer CoPPIX, which has no iron in its composition and does not induce oxidative stress as a provocation [39], whereas in the former, the HO-1 inducer heme acts by first causing oxidative stress and then activating antioxidant defenses [89], besides being a source of iron to the parasite.

**Fig 2:**
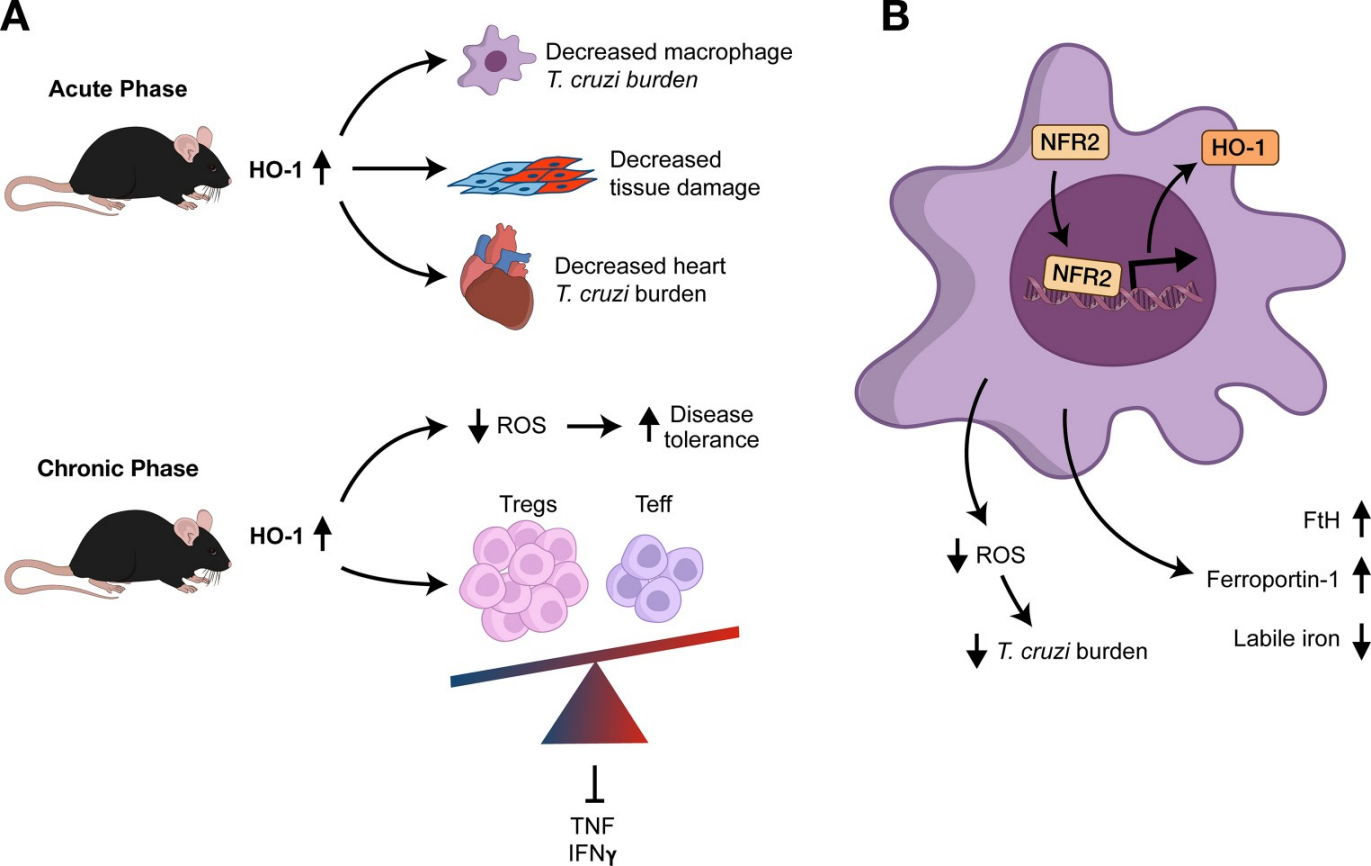
HO-1 in Chagas disease. (A) HO-1 promotes increased resistance in acute *T*. *cruzi* infection in mice. HO-1 expression reduces macrophage and heart parasitism and is associated with increased resistance to the infection during acute and chronic phases of the disease. (B) Up-regulation of HO-1 expression in macrophages reduces intracellular ROS and labile iron pool through FtH and ferroportin-1 expression. The net result is reduction of parasitism. FtH, ferritin H chain; HO-1, heme oxygenase 1; IFN, interferon; NRF2, nuclear factor erythroid 2-related factor 2; ROS, reactive oxygen species; Teff, T effector cells; TNF, tumor necrosis factor; Treg, regulatory T cell.

Although the increase in the inflammatory response after HO-1 inhibition by ZnPPIX was beneficial after 9 days post infection to promote nitric oxide production and parasite control, it was followed by a significant increase in tumor necrosis factor alpha (TNF-α) and interferon gamma (IFN-γ) in different tissues. These cytokines are crucial players in the anti-chagasic immune response at the acute phase and also reported to promote tissue damage [90–92]. HO-1 inhibition by ZnPPIX has also been demonstrated to reduce the number of regulatory T cells (Tregs) (CD4^+^ CD25^+^ T cells) in cardiac tissue [93]. In fact, HO-1 increases Treg–T-effector cell ratio, mediating tissue protection against intestinal and lung inflammation [94–96], and cardiac tissue protection [93, 97]. In contrast to the inhibition of HO-1 by ZnPPIX, its induction by heme administration increased the survival of *T*. *cruzi*–infected mice due to an anti-inflammatory response and consequent tissue protection. Overall, these studies highlighted the protective role of HO-1 induction in Chagas disease animal models. However, in contrast to malaria models, HO-1 has a deep impact on parasitemia reduction and host resistance to infection. It is important to note, however, that HO-1 also positively impacts host disease tolerance during *T*. *cruzi* infection by reducing inflammation-derived damage (Fig 2).

## HO-1 in leishmaniasis

Leishmaniasis is caused by parasites from the genus *Leishmania*. In mammalian hosts, *Leishmania* is an obligate intracellular protozoan parasite of macrophages that replicates within phagolysosomes with more than 21 species reported to cause disease in humans worldwide [98]. The disease presents 3 major forms: the cutaneous form (subdivided into localized, diffuse, and disseminated forms), the muco-cutaneous form, and the visceral form; the latter causes the majority of the 20,000 annual deaths associated with the disease [99]. Although some *Leishmania* species are associated with certain forms of the disease, host endogenous factors also contribute to govern the clinical presentation and severity of the disease.

*Leishmania* sp. is closely related to *T*. *cruzi* and shares some biological features with this parasite, such as the lack of biosynthetic route to generate heme [100]. Since *Leishmania* sp. depends on host heme for several metabolic pathways, up-regulating HO-1 expression could represent a strategy to inhibit intracellular parasite growth by reducing heme availability. The first study to evaluate the role of HO-1 on leishmaniasis, using *L*. *mexicana pifanoi*, showed that amastigotes induce high HO-1 expression early after macrophage infection [55]. Inhibition of HO-1 activity with SnPPIX increased ROS production after amastigote phagocytosis, while induction of HO-1 with CoPPIX reduced the amounts of superoxide, an important ROS associated to *Leishmania* control inside macrophages [101]. However, these authors did not study the effects of manipulating HO-1 on parasite burden. From this study, it became clear that one of the mechanisms that govern the reduction of ROS levels by HO-1 is decreased heme availability, which abolishes gp91^*phox*^ maturation, the heme binding subunit of peroxide generating NADPH oxidase [102], within the parasitophorus vacuole [49]. Interestingly, gp91^*phox*^ genetic deletion affects the immune response to *L*. *major*, resulting in visceralization [103]. In addition, mice deficient in gp91^*phox*^ have increased parasitemia and delayed control of infection with *L*. *donovani* [104]. In fact, a recent study demonstrated that infection of macrophages with *L*. *donovani* induces the expression of HO-1, which in turn reduces the amount of heme and the maturation of gp91^*phox*^, resulting in decreased ROS and increased survival of amastigotes [58]. Moreover, the authors demonstrated that inhibition of inflammatory cytokines by HO-1 is dependent on CO through interference of toll-like receptor (TLR) signaling. Inhibition of HO-1 with SnPPIX in mice reduces parasite burden, whereas it increases the amount of IL-12 and TNF [105]. These studies highlight a lower host resistance associated to HO-1 expression during leishmaniasis.

Infection by *L*. *chagasi i*s associated with an increase in HO-1 expression in mouse macrophages. In such a context, CoPPIX (through HO-1 increased expression) promotes the progression of infection, and conversely, macrophages from *Hmox1*^−/−^ mice present lower parasite burden compared to wild-type controls [106]. In agreement with these data, HO-1 induction reduces TNF-α expression and NO production by macrophages stimulated with IFN-γ in vitro, a classical mechanism involved in the control of parasite burden. In dog macrophages, the induction of HO-1 by CoPPIX also correlates with increase *L*. *infantum* amastigotes and reduction of NO and ROS, whereas the inhibition of HO-1 activity decreases the infection burden and increases NO and ROS [107].

Treatment of primary human macrophages with resolvin D1, an important eicosanoid with anti-inflammatory and pro-resolving properties, promotes intracellular *L*. *amazonensis* replication through a mechanism associated with induction of HO-1 [108]. It has been proposed that sand fly saliva increases the infection of Leishmania both in vitro and in vivo by modulating several physiological responses of the vertebrate host [106, 109]. The ability of sand fly saliva to induce NRF2 and HO-1 in human and mice skin in vivo and on macrophages in vitro, inhibiting the inflammatory response, indicates a previously unappreciated mechanism by which vector saliva affects resistance to *Leishmania* infection [109]. A recent study showed that patients with higher concentrations of heme in the serum and increased expression of HO-1 have higher parasite burden and a decreased number of neutrophils [110–111]. These results are corroborated by the observation that human neutrophils stimulated with heme have higher parasite numbers.

**Fig 3:**
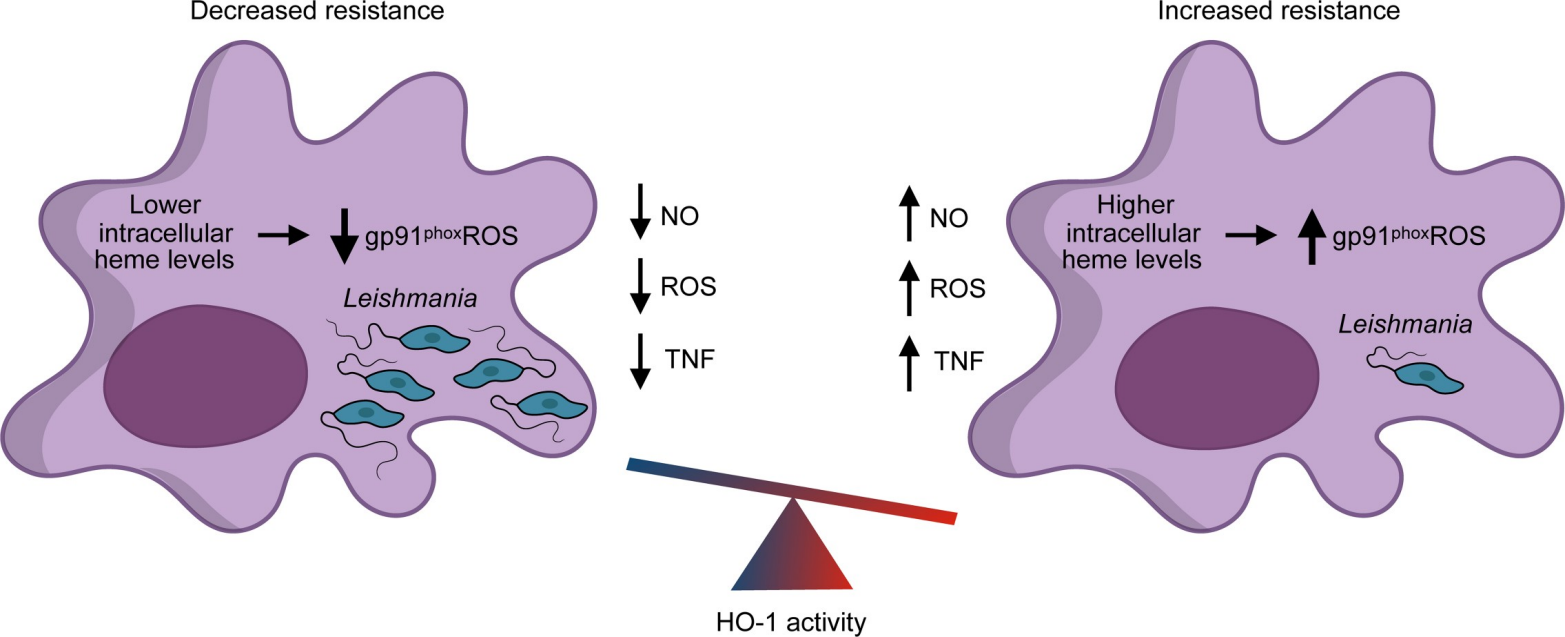
HO-1 on *Leishmania* sp. *Leishmania* infection induces HO-1 on macrophages causing a reduction in intracellular levels of heme that affect gp91Phox activity and ROS generation. NO and TNF production are also inhibited by HO-1. Thus, up-regulation of HO-1 expression increases parasitism and disease tolerance. gp91Phox, glycosylated 91-kDa phagocyte NADPH oxidase; HO-1, heme oxygenase 1; NO, nitric oxide; ROS, reactive oxygen species; TNF, tumor necrosis factor.

Conversely, different compounds with leishmanicidal activities reduce the expression of HO-1 [111–113]. Miltefosine has direct activity against *L*. *donovani* but also contributes to host response against infection in a mechanism that involves HO-1 inhibition [113]. Interestingly, recent studies demonstrated that treatment with plant extracts—precursors of flavonoids or purified flavonoids presenting anti-leishmanicidal activity in promastigotes and amastigotes of *L*. *amazonensis* and *L*. *brasiliensis*—induce NRF2, HO-1, and FtH expression, reducing the labile iron pool of infected macrophages [114–116]. The authors attribute the reduction on macrophage infection by these plant products in part to the depletion of available iron. Geroldinger and colleagues [117] showed that inhibitors of HO-1 could inhibit the leishmanicidal activity of artemisinin. Thus, altogether these results point to a key role of HO-1 in the progression of the disease and indicate the need for more studies evaluating HO-1 as a potential target for therapeutic strategies. In conclusion, HO-1 induction is associated with decreased resistance to *Leishmania* sp. infection, inhibiting important mediators of intracellular clearance of the parasite, such as NO, ROS, and TNF (Fig 3).

## HO-1 in toxoplasmosis

**Fig 4:**
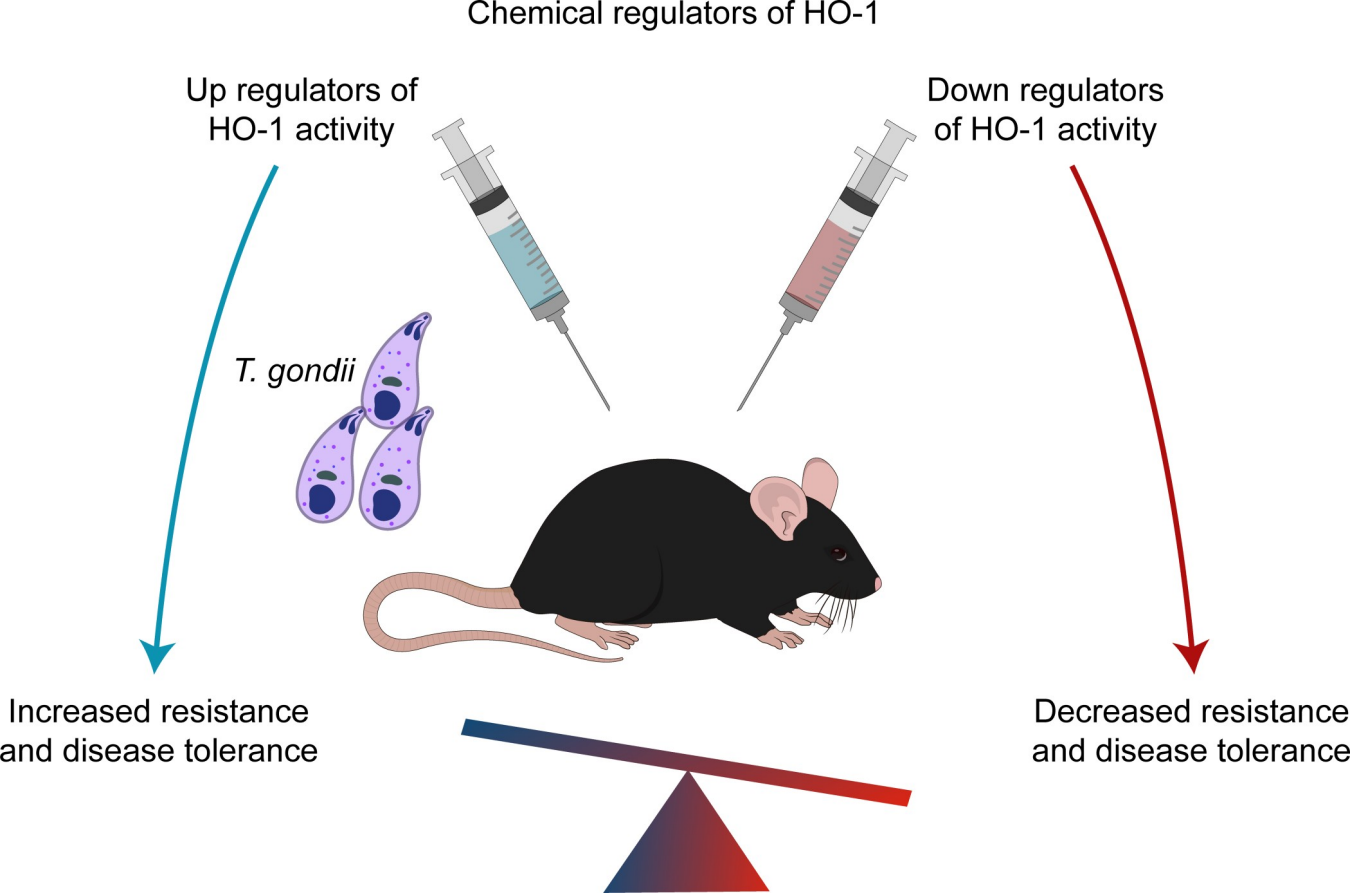
HO-1 on toxoplasmosis. In mice pretreated with chemical regulators of HO-1, induction of HO-1 expression decreases parasite burden on tissues and the morbidity of infected mice, while inhibition of HO-1 activity produced the opposite effect. This indicates that, in toxoplasmosis, HO-1 increases resistance and disease tolerance. HO-1, heme oxygenase 1.

Toxoplasmosis is a disease with worldwide distribution caused by the protozoan parasite *Toxoplasma gondii*. *T*. *gondii* is able to infect almost all mammals, and its importance to humans was evidenced 70 years ago when Schwartzman and colleagues described congenital toxoplasmosis [118]. Infection by *T*. *gondii* is a global health concern especially because of the emergence of AIDS and the increased use of immunosuppressive therapies [119–121]. Similar to other protozoan infections, a cellular immune response is crucial to control and inhibit *T*. *gondii* dissemination, but excessive inflammation is associated with tissue damage and disease [122]. *T*. *gondii* infection induces a robust HO-1 expression in the lungs of BALB/C and C57BL/6 mice [13]. HO-1 inhibition with ZnPPIX resulted in increased tissue parasitism by cyst-like structures and parasitophorous vacuoles. Conversely, treatment with heme, a classic HO-1 inducer, reduced parasite burden. Both treatments increased the expression of TNF and IFN-γ in the lungs, two factors known to play key roles in the immune response to *T*. *gondii*. Two other inducers of HO-1, curcumin and resveratrol, were also associated with an anti-inflammatory protective effect in a model of ileitis after *T*. *gondii* oral infection [123]. Experiments using *Hmox1*^*−/−*^ mice are required to define the putative participation of HO-1 in the protective effects in these models of *T*. *gondii*. Finally, these results indicate HO-1 as a host factor capable of increasing both resistance (Fig 4) and disease tolerance to *T*. *gondii* infection, a scenario that makes HO-1 induction a candidate therapeutic approach to treat patients with toxoplasmosis.

## Therapeutic opportunities

The development of drugs and vaccines to infectious diseases, including parasitic diseases, has been oriented to reduce or eliminate the pathogen load, acting directly in the parasite or increasing the resistance of the host. In recent decades, few new drugs against protozoan parasites have become available, and in the case of antimalarial drugs, the emergence of resistance is a major problem related to controlling the disease. Thus, the development of drugs that increase disease tolerance, aiming at conditions that are caused by the host response to protozoan parasite infections, could be a strategy to complement the current therapies based on antiprotozoal drugs. This is an open and promising field of study and an opportunity to drug development. As discussed earlier, HO-1 exerts differential influences on resistance and disease tolerance in malaria, Chagas disease, leishmaniasis, and toxoplasmosis.

In the case of malaria, the beneficial effects of increased HO-1 expression could also be reproduced by its byproduct, CO. We think that HO-1 inducers or CO-based therapy are good candidates for patients with severe malaria [124]. Considering the neutral or negative impact of HO-1 induction or CO on pathogen load, we speculate that these treatments should be combined to antimalarial drugs, especially during the liver stage of the disease. A similar scenario is observed in leishmaniasis, suggesting that inducers of HO-1 could be used as adjunctive therapy associated to leishmanicidal drugs, controlling tissue damage [125]. In Chagas disease, HO-1 induction seems to be promising, since HO-1 is associated with both increased resistance and disease tolerance. Several compounds that induce HO-1 are beneficial to mice chronically infected with *T*. *cruzi* even when the treatment started after the establishment of severe cardiomyopathy [126–127]. Similarly, HO-1 inducers seem to be good candidates to treat toxoplasmosis, as HO-1 increases both resistance and disease tolerance. Future studies using improved experimental animal models and clinical trials will determine whether some of the promises of manipulating HO-1 on protozoan infections can be fulfilled.

## Conclusions

HO-1 is an essential enzyme regulating heme and iron metabolism, stress, and inflammatory responses. HO-1 induction has been described as an important mechanism of host protection in a number of sterile and infectious disease models in mice [11, 128, 129]. In protozoan diseases, HO-1 might influence both resistance and disease tolerance. HO-1 increases disease tolerance without interfering with the parasite burden during the erythrocytic phase of malaria. However, HO-1 can increase parasite burden during the hepatic phase of malaria and in leishmaniasis, thus reducing resistance to infection. Conversely, in Chagas disease and toxoplasmosis, HO-1 exerts protection by increasing resistance or reducing parasite growth. The mechanisms by which HO-1 exerts its protective effects on pathology are associated with its anti-oxidative and anti-inflammatory effects, which restrict cell death and tissue damage caused by inflammatory response and oxidative stress. Based on the previously discussed findings, we assume that HO-1 expression can influence protozoan infections by a number of mechanisms, including the reduction of heme availability to pathogens, especially from the Trypanosomatidae family; altering iron metabolism; regulating the immune response (direct influence on resistance and disease tolerance to inflammatory damage); restraining the pathogen load (through its influence on microbicidal molecules such as NOX2); and ameliorating pathogen and inflammatory mediated damage (disease tolerance), through its cytoprotective effects. The outcome of the manipulation of HO-1 expression and activity reflect the balance between its effects on resistance and on disease tolerance during protozoan infections. In these complex disease settings, understanding the precise role of HO-1 appears a difficult, but very promising, task.
